# Efficient Production of 2,5-Diketo-D-gluconic Acid by Reducing Browning Levels During *Gluconobacter oxydans* ATCC 9937 Fermentation

**DOI:** 10.3389/fbioe.2022.918277

**Published:** 2022-07-08

**Authors:** Guang Li, Xiaoyu Shan, Weizhu Zeng, Shiqin Yu, Guoqiang Zhang, Jian Chen, Jingwen Zhou

**Affiliations:** ^1^ Science Center for Future Foods, Jiangnan University, Wuxi, China; ^2^ Key Laboratory of Industrial Biotechnology, Ministry of Education and School of Biotechnology, Jiangnan University, Wuxi, China; ^3^ Engineering Research Center of Ministry of Education on Food Synthetic Biotechnology, Jiangnan University, Wuxi, China; ^4^ Jiangsu Province Engineering Research Center of Food Synthetic Biotechnology, Jiangnan University, Wuxi, China

**Keywords:** detection method, 2,5-diketo-D-gluconic acid, browning, *Gluconobacter oxydans* ATCC 9937, feed-batch

## Abstract

D-Glucose directly generates 2-keto-L-gulonic acid (2-KLG, precursor of vitamin C) through the 2,5-diketo-D-gluconic acid (2,5-DKG) pathway. 2,5-DKG is the main rate-limiting factor of the reaction, and there are few relevant studies on it. In this study, a more accurate quantitative method of 2,5-DKG was developed and used to screen *G. oxydans* ATCC9937 as the chassis strain for the production of 2,5-DKG. Combining the metabolite profile analysis and knockout and overexpression of production strain, the non-enzymatic browning of 2,5-DKG was identified as the main factor leading to low yield of the target compound. By optimizing the fermentation process, the fermentation time was reduced to 48 h, and 2,5-DKG production peaked at 50.9 g/L, which was 139.02% higher than in the control group. Effectively eliminating browning and reducing the degradation of 2,5-DKG will help increase the conversion of 2,5-DKG to 2-KLG, and finally, establish a one-step D-glucose to 2-KLG fermentation pathway.

## Introduction

L-Ascorbic acid has important roles in food (Cendrowski et al., 2021), medicine (Pardo-Planas et al., 2017), and health (Anitra and Silvia, 2017; Reddy et al., 2020). Recent research has also suggested it has particular functions in electron transfer (Xin et al., 2022) and microbial fuel cells (Islam et al., 2020). Currently, in industry, L-ascorbic acid is mainly synthesized by 2-keto-L-gulonic acid (2-KLG) *via* a simple chemical reaction, which uses D-sorbitol as a substrate (Cai-Yun Wang et al., 2018; Zhang et al., 2018; Zeng et al., 2020). In addition, 2-KLG can also be directly generated by 2,5-diketo-D-gluconic acid (2,5-DKG) *via* a simple reduction reaction (Sonoyama et al., 1982). 2,5-DKG is commonly and directly synthesized using D-glucose as a substrate. In addition to 2,5-DKG being the precursor of 2-KLG, recent studies reported its synthesis may also increase rare-earth element recovery in wastewater (Jindra et al., 2016; Schmitz et al., 2021). As 2,5-DKG reactions during synthesis and decomposition processes are unclear, further research in these areas may provide insights on one-step 2-KLG synthesis and environmental protection.

In *Erwinia* sp. (Sonoyama et al., 1988), *G. oxydans* (Ji and Gao, 2001), *Pseudomonas putida* (Nikel and de Lorenzo, 2018), and *Tatumella citrea* (previously known as *Erwinia citrea*) (Andreeva et al., 2011; Marin-Cevada and Fuentes-Ramirez, 2016), D-glucose is gradually oxidized to D-gluconic acid (GA), 2-keto-D-gluconic acid (2-KG), and 2,5-DKG *via* glucose dehydrogenase (GDH), D-gluconic acid dehydrogenase (GADH), and 2-keto-D-gluconic acid dehydrogenase (2-KGDH), respectively, on the cell membrane or in the periplasmic space (Kataoka et al., 2015). Then, 2,5-DKG can be transformed into 2-KLG by 2,5-DKG reductase from *Corynebacterium glutamate* (Badwar et al., 2020). Based on the reported synthesis and metabolism of 2,5-DKG, a new one-step fermentation route was established for 2-KLG production directly from D-glucose, using recombinant *Erwinia herbicola* encoding the 2,5-DKG reductase gene from *Corynebacterium* sp. (Anderson et al., 1985). As one of the cheapest carbon sources, the use of D-glucose for 2-KLG production effectively solves the disadvantages of high energy consumption, high water consumption, long cycle, and difficulty in accurate control in other pathways. However, due to uncharacterized speed-limiting factors, 2,5-DKG pathway research is still in its infancy (Panpan Wang et al., 2018).

The main speed-limiting issue with the 2,5-DKG pathway is the transformation from 2,5-DKG to 2-KLG. This reaction is mainly catalyzed by 2,5-DKG reductase; therefore, generating a highly catalytically efficient 2,5-DKG reductase could facilitate a one-step fermentation system. Previous studies have investigated different strategies to strengthen 2,5-DKG conversion to 2-KLG, and increase 2-KLG production from D-glucose *via* the 2,5-DKG pathway; however, the effect is not obvious (Scott et al., 2002; Jeudy et al., 2006; Kaswurm et al., 2013).

In addition to generate an efficient 2,5-DKG reductase, it is important to select a good chassis strain (Vivek et al., 2021), optimize 2,5-DKG production processes, and identify possible speed-limiting factors. Previous studies investigated 2,5-DKG production improvements. A mixed culture of two bacteria from soil was developed to convert 50 g/L D-glucose to 2,5-DKG, and generated a 92% conversion ratio (Sulo et al., 2001). In addition, 65.8 g/L 2,5-DKG was harvested from a 10-L bioreactor using a high-performance liquid chromatography (HPLC) method (Qazi et al., 1991). However, the fermentation time in these studies is relatively long, generally more than 120 h. According to our previous studies, due to the lack of standards and the deviation of detection methods, these research data may have large errors. Therefore, the development of a relatively accurate quantitative method monitoring 2,5-DKG changes is warranted.

In this study, we established a quantitative detection method for 2,5-DKG based on pure enzyme catalysis. Using this method, we compared 2,5-DKG production levels in five strains, with *G. oxydans* ATCC 9937 selected as the optimum strain. After analyzing *G. oxydans* ATCC 9937-mediated 2,5-DKG production from D-glucose, a putative speed-limiting factor was identified. By knocking out and overexpressing the gene encoding the putative rate-limiting enzyme, we identified browning issues, caused by prolonged fermentation times, as the main factor leading to low 2,5-DKG accumulation. While browning is irreversible, it can be slowed down by shortening fermentation times. To reduce 2,5-DKG production losses caused by browning, we accelerated strain growth and reduced fermentation times by optimizing D-glucose addition at the start of fermentation. Finally, the browning degradation of 2,5-DKG was reduced and production generated a 139.02% increase using the feeding mode. This study provides a basis for further improving the one-step fermentation of D-glucose to produce 2-KLG.

## Materials and Methods

### Genes, Plasmids, and Strains

*Escherichia coli* BL21 (DE3) and vector pET28a were obtained from Sangon Biotech Corporation (Shanghai, China), and used to express 2,5-DKG reductase. *C. glutamicum* ATCC 13032 was purchased from the American Type Culture Collection (ATCC), and used to amplify the gene of 2,5-DKG reductase. The 2,5-DKG reductase gene from *C. glutamicum* ATCC 13032 was amplified with primer pair *dkg*-F and *dkg*-R. The pk18mobsacb vector was used to knock out *kgdSLC* gene. The plasmid pBBR1MCS-2 is used as a vector to overexpress *kgdSLC* gene.

*G. oxydans* ATCC 9937, *P. putida* ATCC 21813 and *C. glutamicum* ATCC 13032 were purchased from the American Type Culture Collection (ATCC); *Tatumella citrea* CICC 10802 and *T. citrea* CICC 10803 strains were obtained from the China Center of Industrial Culture Collection (Beijing, China); *P. putida* KT 2440 was preserved in our laboratory. All these strains were cultured in fermentation medium to verify their 2,5-DKG yield.

All primers are list in Table 1.

**TABLE 1:** Primers used in this study.

Primers	Sequence (5′-3′)
*dkg*-F	CAT​CAT​CAT​CAT​CAT​CAC​ATG​GAT​CAG​AAG​AAT​AAG​CTT​TCG​AAG​TCT​GA
*dkg*-R	GCT​TCC​TTT​CGG​GCT​TTG​CTA​GTT​CAGA​TCA​TTC​GGG​TGT​GAA​CC
pET28a-F	GTG​ATG​ATG​ATG​ATG​ATG​GCT​GCT​G
pET28a-R	CAA​AGC​CCG​AAA​GGA​AGC​TGA​G
*kgdC* up-F	ATT​CGA​GCT​CGG​TAC​CCG​GGCCC​ATT​CCA​CAG​GGA​CGG​AG
*kgdC* up-R	CTT​TCA​GAC​CGA​GCG​CTG​CC
*kgdC* down-F	GGC​AGC​GCT​CGG​TCT​GAA​AGGCC​TGG​ATT​TGG​GCC​AGG​TT
*kgdC* down-R	CCT​GCA​GGT​CGA​CTC​TAG​AGCCG​TCC​TTG​CTT​AAC​TGG​ATA​T
*kgdS* up-F	ATT​CGA​GCT​CGG​TAC​CCG​GGCGA​GCG​TCC​TGT​CGA​ATT​GC
*kgdS* up-R	TTC​CGC​AAC​AGA​GAT​CAT​TGT​CAT​TT
*kgdS* down-F	CAA​TGA​TCT​CTG​TTG​CGG​AAGCT​CAG​TCG​AGG​ACA​GAA​CG
*kgdS* down-R	CCT​GCA​GGT​CGA​CTC​TAG​AGGCC​CCG​ATG​GGG​CAG​ATG​GG
*kgdL* up-F	ATT​CGA​GCT​CGG​TAC​CCG​GGCAA​CCC​ATT​CTG​GTC​GAA​CGT
*kgdL* up-R	GGG​TTA​GTG​CTC​CTG​ACG​GG
*kgdL* down-F	CCC​GTC​AGG​AGC​ACT​AAC​CCAGG​ACT​GGA​TGC​GGT​CAA​CA
*kgdL* down-R	CCT​GCA​GGT​CGA​CTC​TAG​AGTCA​CAT​TCG​GCG​CAT​ACC​AT
pk1-R	CCC​GGG​TAC​CGA​GCT​CGA​AT
pk1-F	CTC​TAG​AGT​CGA​CCT​GCA​GGC​A
pBBR-*kgdSLC*-F	TCA​GAA​AAG​GAA​GAA​TAA​CAGAA​ATG​ACA​ATG​ATC​TCT​GTT​GCG​G
pBBR-*kgdSLC*-R	ACT​AAA​GGG​AAC​AAA​AGC​TGTCA​GGC​CTT​CGC​CTT​TCG​GG
P7-F	ACT​CAC​TAT​AGG​GCG​AAT​TGCAC​GAT​CGT​CGG​CAA​AGT​GA
P7-R	TGT​TAT​TCT​TCC​TTT​TCT​GAT​CGT​GAC​GA
pBBR-F	CAA​TTC​GCC​CTA​TAG​TGA​GTC​GTA​TTA​CG
pBBR-R	CAG​CTT​TTG​TTC​CCT​TTA​GTG​AGG​GT

### Media and Culture Condition

D-Gluconic acid, 2-KG, 5-keto-D-gluconic acid (5-KG), and 2-KLG were purchased from Toronto Research Chemicals INC. (Toronto, Canada). An Aminex HPX-87H liquid chromatographic column was purchased from Bio-Rad Laboratories INC. (CA, United States). Yeast extract was purchased from Oxoid (Hampshire, United Kingdom).

D-Sorbitol medium preparation: D-sorbitol (50 g/L), yeast extract (10 g/L), KH_2_PO_4_ (1 g/L), MgSO_4_ (0.25 g/L), and 20 g/L agar were added to solid medium. Fermentation medium: 100 g/L D-glucose, 10 g/L yeast extract, and 0.25 g/L MgSO_4_.

*G. oxydans* ATCC 9937 was grown on D-sorbitol solid medium for 20 h at 30°C, after which a single colony was transferred to liquid D-sorbitol medium and cultivated for 24 h at 30°C as seeding, and then was transferred to fermentation medium. *T. citrea* CICC10802, *T. citrea* CICC10803, *P. putida* ATCC21812, *P. putida* KT2440, and *E. coli* BL21 were activated in a solid LB medium at 30°C for 16 h and then transferred to a liquid LB medium at 30°C for 16 h, after which was transferred to fermentation medium.

Batch fermentation was performed in a 5-L fermenter (T&J-B type, J Bio-engineering Co., Ltd, Shanghai, China). The stirring speed gradually increased from 300 to 500 rpm and the ventilation ratio was 1 vvm. A pH of 4.9 was automatically maintained by adding 6 mol/L NaOH.

### Preparation Method of 2,5-DKG

The fermentation broth was collected and centrifuged to remove bacteria. Used HyperSep Retain-AX solid-phase extraction column (ThermoFisher Scientific, Healthpoint, United States) to remove some protein impurities and pigments in the supernatant, and then lyophilized and stored, and dissolved in water after use.

### Overexpression and Purification of 2,5-DKG Reductase

*E. coli* BL21 (DE3) cells expressing the recombinant plasmid pET28a-Histag-2,5-DKGR were cultured at 220 rpm for 10 h at 37°C. Then, 500 µL of the culture was transferred to TB medium and grown at 220 rpm at 37°C. When the OD_600_ reached 0.8, the temperature was reduced to 20°C, 0.1 mM isopropyl β-d-1-thiogalactopyranoside was added, and incubation continued for 16 h. Then, cells were harvested by centrifugation at 8,000 *g* for 5 min. After washing twice in phosphate buffer saline (PBS), cells were disrupted using a high-pressure cell cracker, and the crude enzyme solution was clarified by centrifugation (Lei et al., 2019). The solution was purified using a protein purifier, quick-frozen in liquid nitrogen, and stored at −80°C.

### Catalytic Studies on 2,5-DKG Reductase

We prepared different concentrations of 2,5-DKG. Then, added 100 µL 2,5-DKG reductase and excess NADPH, supplemented the volume to 1 ml with Tris-HCl (pH 7.0), and incubated samples at 30°C for 4 h to ensure the substrate was completely transformed to 2-KLG. (Scott et al., 2002). Then, the concentration of 2-KLG in the system was detected by HPLC. In the first reaction, a small amount of 2,5-DKG and sufficient NADPH were added, and reacted for more than 4 h to ensure that 2,5-DKG is completely converted into 2-KLG. Then, the concentration of 2,5-DKG was gradually increased, and the NADPH to be added was estimated according to the results of the first reaction, so as to ensure that the cofactor is always sufficient or excessive to ensure the complete conversion of each time.

**FIGURE 1:**
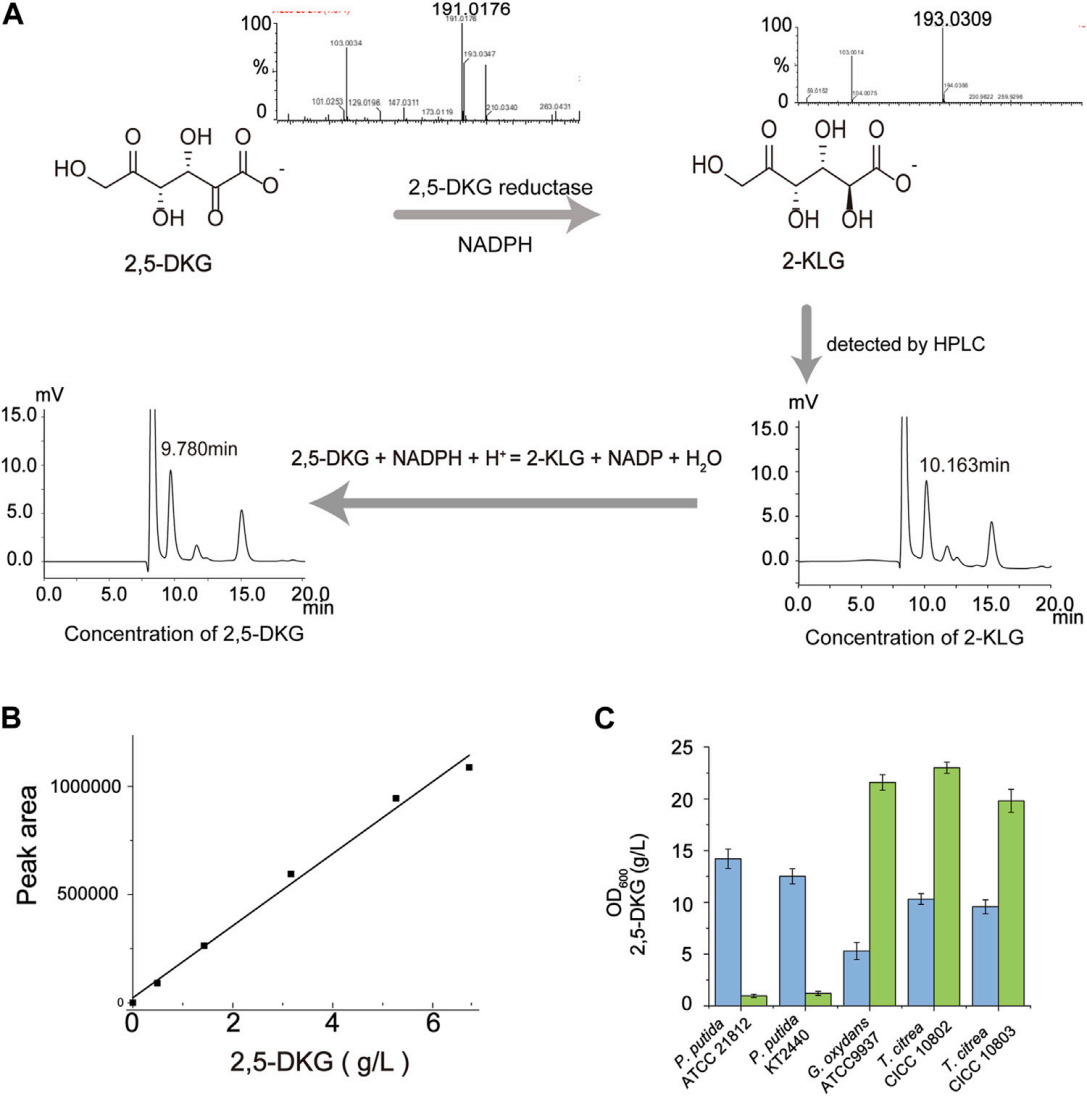
Quantification of 2,5-DKG by indirect HPLC analysis. **(A)** The peak time of 2,5-DKG is different from that of 2-KLG in HPLC detection. Therefore, 2,5-DKG which is difficult to quantify can be transformed into 2-KLG which is easier to quantify, and the real concentration of 2,5-DKG can be calculated from the concentration of 2-KLG. **(B)** Standard 2,5-DKG curve. **(C)** Comparing 2,5-DKG production in different strains. *P. putida* ATCC 21812 (No production); *P. putida* KT 2440 (No production); *G. oxydans* ATCC 9937 (21.6 g/L); *T. citrea CICC* 10802 (23.6 g/L); and *T. citrea* CICC 10803 (19.8 g/L). Blue = OD_600_; green = 2,5-DKG concentrations.

To verify the accuracy of the standard curve, we fitted the calculated 2,5-DKG concentration to the HPLC peak area (Figure 1B), and the relationship between the two was determined as follows:

y=160151.56x+14403.69 (1)

where x = the 2,5-DKG concentration and y = the peak area; therefore, the *R* ^*2*^ value of the relationship = 0.9924, indicating relatively high accuracy of the detection method (Figure 1B).

### Knockout and Overexpression of *kgdSLC* Gene

The pk18mobsacb vector containing SacB system, previously developed in our laboratory, was used to edit genes in the *G. oxydans* ATCC 9937 strain (Chen et al., 2021). Using the *G. oxydans* ATCC 9937 genome as a template, primers were designed to amplify 1000 bp homologous arms upstream and downstream of the *kgdS*, *kgdL* and *kgdC* for PCR knockout (Table 1). Then, upstream and downstream homologous arms of the target gene were recombined using the pK18mobsacb vector to generate a recombinant plasmid. After verification, the plasmid was transformed into *G. oxydans* ATCC 9937. For more detailed procedures, please refer to our previous study (Qin et al., 2021).

Using the *G. oxydans* ATCC 9937 genome as a template, primer pair pBBR-*kgdSLC*-F and pBBR-*kgdSLC*-R were designed to amplify *kgdSLC*, primer pair P7-F and P7-R were designed to amplify promoter sequence. Then, *kgdSLC* and promoter sequence were recombined using the pBBR1MCS-2 vector to generate a recombinant. After verification, the plasmid was transformed into *G. oxydans* ATCC 9937 to overexpress *kgdSLC*.

### Biomass and High-Performance Liquid Chromatography Analysis

To avoid fermentation broth interference in terms of color generation for spectrophotometric assay, bacteria were centrifuged, resuspended in an equal volume of PBS, and OD_600_ values were recorded.

D-Gluconic acid, 2-KG, 2,5-DKG, and 2-KLG levels were detected and quantified by HPLC using a Shimadzu LC-20A system equipped with a refractive index detector on an Aminex HPX-87H column at 40°C in 5 mmol/L H_2_SO_4_ eluent (0.5 ml/min = flow rate).

2,5-DKG and 2-KLG were measured using LC-MS under the conditions of Table 2.

**TABLE 2:** Detection conditions of LC-MS.

Instrument	Waters Quattro Premier XE	
Ion mode	ESI−	ESI+
Capillary	3.0 KVolts	3.5 KVolts
Cone	20/50 V	
Source block temp	100°C	
Desolvation temp	400°C	
Desolvation gas flow	700 lit/hr	
Cone gas flow	50 lit/hr	
Collision energy	6 eV	
Mass range	50–2000 m/z	
Detector	1800 V	

### Browning Measurement by Ultraviolet Spectrophotometer

Samples were taken every 12 h to detect the content of 2,5-DKG and the degree of browning. The browning of fermentation broth was reflected by the absorbance value at 420 nm (Zhao et al., 2017; Tavares et al., 2018).

## Result

### Identifying an Optimum 2,5-DKG-Producing Strain Using Enzyme-Based Detection

The traditional host strain as a one-step fermentation pathway from D-glucose to 2-KLG has been developing slowly. Therefore, we plan to re-screen the appropriate chassis strain and optimize its ability to produce 2,5-DKG. To obtain such a high producer of 2,5-DKG, an effective detection method of 2,5-DKG is required to be established. 2,5-DKG has no standard and lacks quantitative basis, but its oxidation product 2-KLG can be easily quantified. In this study, 2,5-DKG was transformed into 2-KLG by 2,5-DKG reductase, and the actual content of 2,5-DKG was calculated by quantitative analysis of 2-KLG. Firstly, we compared the peak time difference between 2,5-DKG and 2-KLG in HPLC. The peak time of the two were 9.35 and 10.16 min, respectively. Then, the compound 2,5-DKG was completely converted to 2-KLG under the presence of excess cofactor NADPH (Figure 1A); the substrate 2,5-DKG and product 2-KLG were detected by LC-MS. After the concentration of 2-KLG in the solution is detected, the actual concentration of 2,5-DKG in the solution is calculated according to 100% conversion. To verify the accuracy of this method, a standard curve was produced for the 2,5-DKG quantification based on the calculation of molar conversion (Figure B), and the results show that the detection method is reasonable.

Based on this quantitative method, five strains producing 2,5-DKG were compared (Figure 1C), including *G. oxydans* ATCC 9937, *T. citrea* CICC 10802, *T. citrea* CICC 10803, *P. putida* ATCC 21813, and *P. putida* KT 2440. After comparing the yield of 2,5-DKG and OD_600_ of different strains, *G. oxydans* ATCC 9937 was identified as having the highest 2,5-DKG production (4.08 g/L/OD_600_) and was chosen as the starting strain. The strain produced 21.6 g/L 2,5-DKG from 100 g/L D-glucose, with a conversion rate of 20.7%. The conversion rate of *G. oxydans* ATCC9937 from D-glucose to 2,5-DKG is not high. In order to analyze the reasons for the low conversion rate, a detailed understanding of the whole metabolic process and conversion rate of *G. oxydans* ATCC9937 synthesizing 2,5-DKG with D-glucose as the substrate are necessary.

### Metabolites in the Fermentation Process of 2,5-DKG by *G. oxydans* ATCC 9937

**FIGURE 2:**
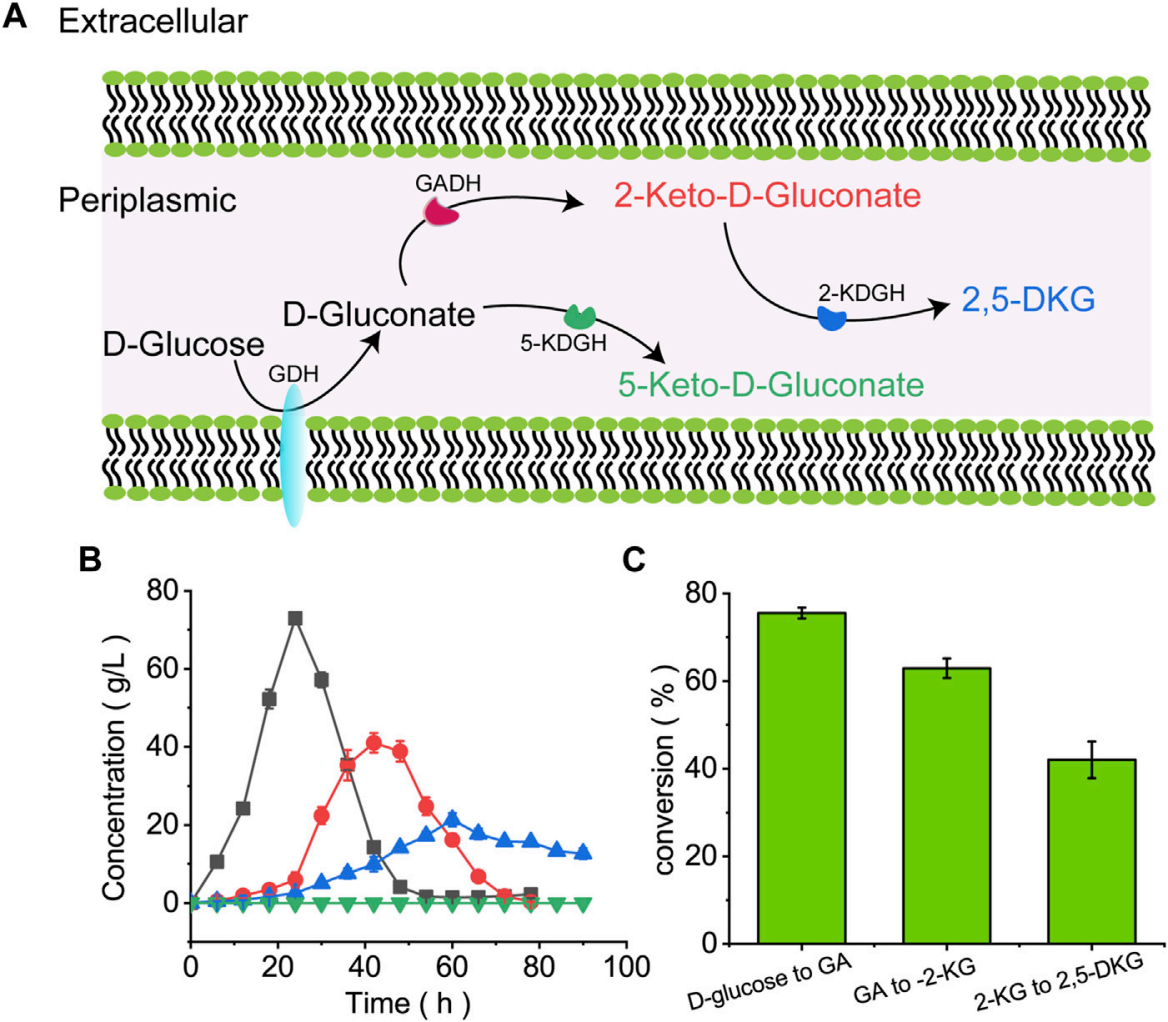
The microbial pathway showing D-glucose conversion to 2,5-DKG. **(A)** D-Glucose conversion to 2,5-DKG in the periplasmic space; **(B)** Consumption and formation of D-gluconic acid, 2-KG, 2,5-DKG, and 5-KG by *G. oxydans* ATCC 9937. Black = D-gluconic acid levels; red = 2-KG levels; blue = 2,5-DKG levels; green = 5-KG levels. **(C)** Illusion rate of various substances.

In *G. oxydans*, D-glucose is oxidized to GA by membrane-bound GDH, then further oxidized to 2-KG or 5-KG by GADH or glycerol dehydrogenase (Qazi et al., 1991) (Figure 2A). As only 2-KG is oxidized to 2,5-DKG by 2-KGDH. To find out the possible rate-limiting factors, this study verified its ability to produce 2,5-DKG. Through the detection of samples in different fermentation cycles, the product of *G. oxydans* ATCC9937 in the fermentation medium with D-glucose as the carbon source is shown in Figure 2B. This study did not detect 5-KG in the medium, it was speculated that the process of *G. oxydans* ATCC 9937 did not or only produce a small amount of 5-KG.

*G. oxydans* ATCC 9937 consumed D-glucose in the first 24 h to generate GA and low 2-KG and 2,5-DKG levels. D-gluconic acid accumulation peaked at 24 h, about 72.96 g/L, and the conversion of D-glucose to GA is about 75.5%. GA was consumed after 52 h, 2-KG peaked at 41.03 g/L at approximately 42 h, and the conversion of GA to 2-KG is about 62.8%. 2,5-DKG peaked at 21.3 g/L at approximately 60 h, and then levels of 2,5-DKG gradually decreased to 12.7 g/L at 90 h. Although the results showed that 2-KG was completely consumed in about 68 h, the conversion of 2-KG to 2,5-DKG is only 41.4% (Figure 2C), which is relatively low. The reason for this phenomenon may be an important rate-limiting factor for the accumulation of 2,5-DKG.

### Knockout and Overexpression of *kgdSLC*

**FIGURE 3:**
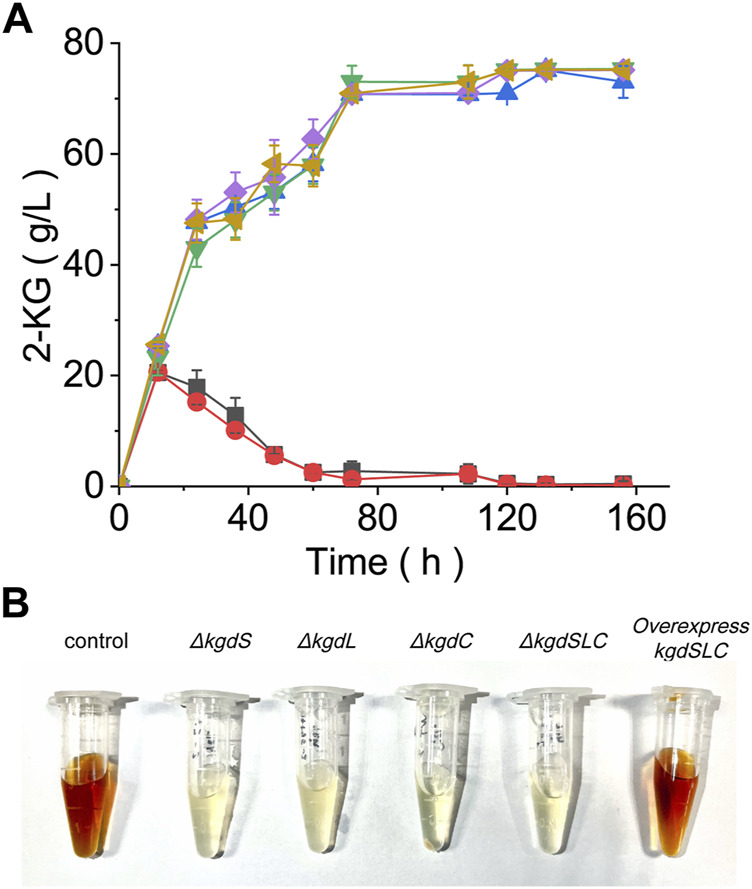
Evaluating the fermentation performance of the *kgdSLC* knockout strain. **(A)** Changes in 2-KG concentrations in fermentation broth after *kgdSLC* knockout and overexpression. Green = *kgdL* knockout. Yellow = *kgdS* knockout. Blue = *kgdC* knockout. Purple = combined knockout. Red = Overexpressed *kgdSLC*. Black = control cells. **(B)** Broth color changes after *kgdSLC* knockout. Regardless of single or combined knockout, the broth no longer turned brown; when *kgdSLC* was overexpressed, the color of the fermentation broth turned brown.

To block the synthesis of 2,5-DKG and find out other possible transformation pathways of 2-KG and optimize them, it is necessary to edit the coding gene of 2-KGDH. The gene sequence encoding 2-KGDH in *G. oxydans* ATCC9937 was found by blast. 2-KGDH contains three subunits, *kgdS*, *kgdL,* and *kgdC* (Shinagawa et al., 2014; Kataoka et al., 2015). In order to further determine that the sequence is correct, we knocked out the three subunits respectively. The result showed that, when compared with original control bacteria, all knockouts failed to convert 2-KG to 2,5-DKG. Until 156 h of fermentation, 2-KG concentrations remained at about 75.1 g/L (Figure 3A); the figure showed that the true conversion rate of GA to 2-KG is about 87.8%, which is much higher than the value we calculated before. No other substances were detected other than 2-KG.

In the absence of side reaction, the low efficiency of 2-KG to 2,5-DKG may also be caused by the low activity or expression of 2-KDGH. So, we used pBBR1MCS-2 plasmid to complement *kgdSLC* gene, and replaced the p7 promoter with higher strength to improve the copy number of *kgdSLC*. Then 2-KG can be transformed into 2,5-DKG again, but 2,5-DKG production did not change significantly when compared with the original control bacteria. Thus, the catalytic efficiency of 2-KGDH did not change significantly whether *kgdSLC* was overexpressed or not. The conversion rate of D-glucose to 2-KG was as high as 76.3%, while the final conversion rate to 2,5-DKG during fermentation was only 23.1%. However, the knockout and overexpression results of *kgdSLC* showed that the main rate-limiting factor of the reaction maybe is not the conversion of 2-KG. The knockout and overexpression of *kgdSLC* also brought another phenomenon; when compared with the yellowish color of the fermentation broth of control cells, the experimental group, which no longer produced 2,5-DKG, was always clear (Figure 3B). In control cells, with increased fermentation time, broth color gradually deepened and finally changed to dark brown, similar to soy sauce. In the earliest relevant studies, it is always mentioned that the production of 2,5-DKG is always accompanied by the color change in fermentation broth. But this color change is more like browning in Maillard reaction. It is necessary to study whether the color change is caused by the browning of 2,5-DKG or other factors.

### Non-Enzymatic Browning is the Main Factor Leading to the Degradation of 2,5-DKG

**FIGURE 4:**
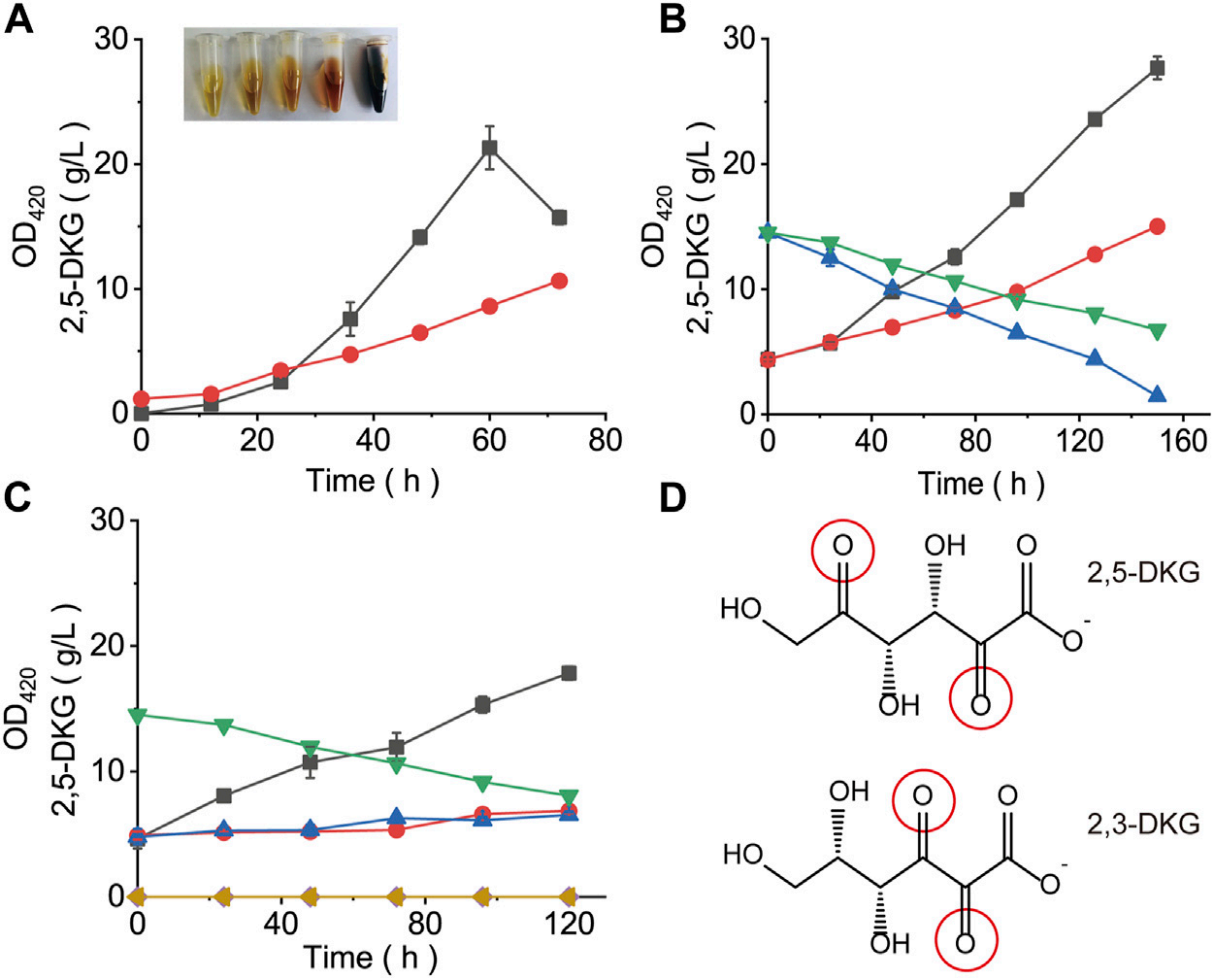
Browning formation (absorbance at 420 nm) and the concentration of 2,5-DKG under different conditions. **(A)** Concentration of 2,5-DKG and absorbance at 420 nm in fermentation broth. Black = 2,5-DKG concentration. Red = Absorbance at 420 nm. With increased fermentation time, 2,5-DKG levels gradually decreased, and the degree of browning gradually increased. **(B)** 2,5-DKG concentration and absorbance at 420 nm in broth plus glutamine. Red = OD_420_ in control cells without glutamine. Black = OD_420_ in the experimental group plus glutamine. Green = 2,5-DKG concentration in control cells. Blue = 2,5-DKG concentration in the experiment group. **(C)** 2,5-DKG concentration and absorbance at 420 nm in broth plus browning inhibitor. Black = OD_420_ in control cells without a browning inhibitor. Blue = OD_420_ in the experimental group plus the browning inhibitor sodium sulfite. Red = OD_420_ in the experimental group plus the browning inhibitor potassium sulfite. Green = 2,5-DKG concentration in control cells. Yellow = 2,5-DKG concentration in the experiment group. **(D)** 2,5-DKG and 2,3-DKG structures.

The OD_420_ value of samples is usually an important index to detect the degree of browning in Maillard reaction (Paravisini and Peterson, 2019). To investigate whether the color change in fermentation broth is related to 2,5-DKG, the OD_420_ values and 2,5-DKG concentrations in fermentation broth at different periods were measured. With increased fermentation time, broth color gradually deepened, and OD_420_ values maintained an increasing trend. Also, 2,5-DKG concentrations maintained an increasing trend in the early stages, but then declined after peaking at 60 h; the concentration was only 74.52% of the maximum value after 72 h (Figure 4A). Then, 2,5-DKG solution was prepared with a concentration of about 15 g/L, and the same quantity of glutamine as 2,5-DKG was added. After this addition, OD_420_ absorbance values increased significantly when compared with control group (2,5-DKG solution without glutamine). After 150 h, 2,5-DKG concentrations in control group decreased by 53.9%, and in the experimental plus glutamine group, it decreased by 90.1% (Figure 4B). By fitting OD_420_ absorbance values of both groups with 2,5-DKG concentrations, we identified a negative correlation between OD_420_ and 2,5-DKG concentrations, but the relationship was not linear.

To further verify that browning is caused by 2,5-DKG, we added a Maillard inhibitor to destroy 2,5-DKG, and then browning will not occur again whether glutamine was added or not (Figure 4C). The current research results showed that the degradation of 2,5-DKG leads to the continuous deepening of the color of fermentation broth, and the main mode of degradation is non-enzymatic browning. This study tried to eliminate or inhibit the browning phenomenon of 2,5-DKG, but it did not work. 2,5-DKG continued browning and degraded with the increase in storage time unless refrigerated. Therefore, in order to reduce the browning of 2,5-DKG and improve the yield of 2,5-DKG, minimizing the fermentation time through optimization is the most feasible and efficient way.

### Enhanced 2,5-DKG Production by Reducing Fermentation Period

**FIGURE 5:**
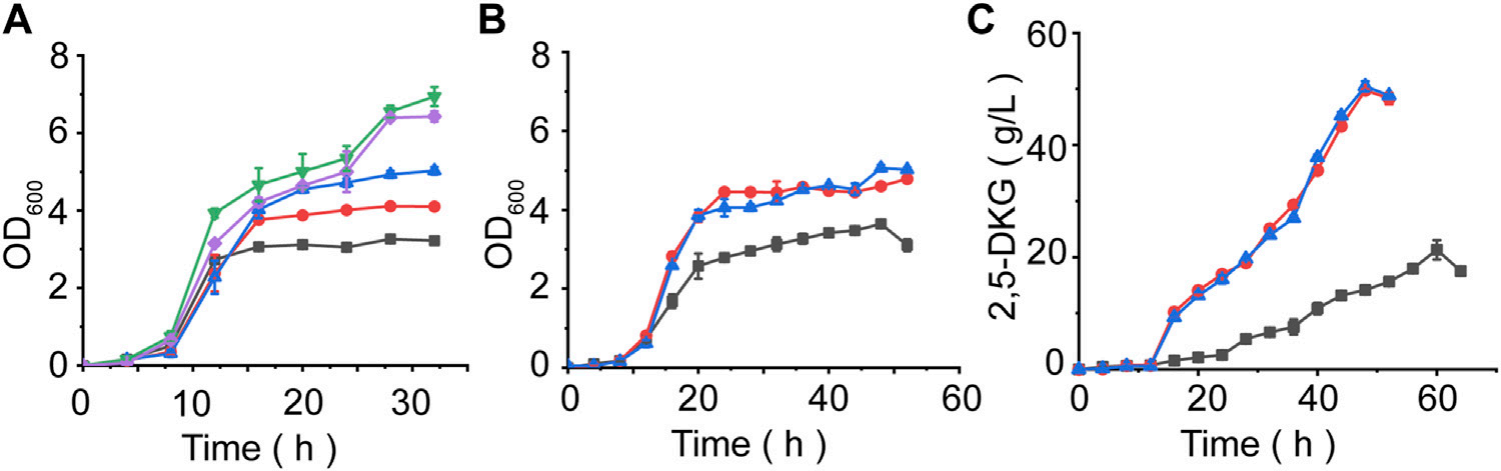
Evaluating fermentation performances under different conditions. **(A)** *G. oxydans* ATCC9937 growth curve at different D-glucose concentrations. Black = 5 g/L D-glucose. Red = 10 g/L D-glucose. Blue = 15 g/L D-glucose. Green = 20 g/L D-glucose. Purple = 25 g/L D-glucose. **(B)** The effect of different feeding methods on OD_600_ values. **(C)** Time course of 2,5-DKG production using different feeding methods. Black = control, initial D-glucose concentration = 100 g/L. Blue: After initial glucose levels were consumed, further glucose was added in a constant-rate feeding mode. Red: Further glucose was added in a single-dose feed-batch mode after initial glucose quantities were consumed.

Inappropriate fermentation conditions will inhibit the growth and metabolism of bacteria, so as to prolong the fermentation time and increase the browning degradation rate of 2,5-DKG. In order to reduce the browning rate and increase the yield of 2,5-DKG, we optimized the fermentation process. In this study, the optimization was carried out by changing the initial D-glucose concentration to reduce the growth inhibition of the strain. To find the best initial D-glucose concentration, we compared the growth curves of strains under different D-glucose concentrations, result showed that the strain grew fastest at an initial D-glucose concentration of 20 g/L (Figure 5A), therefore 20 g/L initial D-glucose concentration was used to optimize the subsequent fermentation process. When the initial D-glucose concentration was reduced to 20 g/L, the ability of the strain to produce 2,5-DKG was significantly accelerated, 12.3 g/L of 2,5-DKG was obtained in 16 h, and the growth of the strain was also significantly accelerated. The biomass of 16 h increased by about 56% compared with the control group (100 g/L D-glucose).

In order to further increase the yield of 2,5-DKG, after the initial D-glucose consumption of 20 g/L is completed, we continued to add D-glucose by feeding. The feeding is carried out in two ways; 1) by adding all the remaining D-glucose at one time, and 2) concentrations were maintained at 20 g/L by feeding to finally reach a concentration of 100 g/L. The results showed that the strains grew rapidly in the first 20 h of fermentation, and began to stabilize after 24 h, whether supplemented or not (feeding began at approximately 16 h). However, after reducing the initial D-glucose concentration, the growth of the strain was significantly better than that of the control group. The OD_600_ of the experimental group was approximately 1.4 times that of the control group, and the final OD_600_ of different feeding methods has little difference (Figure 5B). When compared with control group, 2,5-DKG production peaked after 48 h, and fermentation time had been reduced by approximately 10 h. 2,5-DKG production increased by 139.02% and 133.63%, peaking at 50.9 g/L and 49.7 g/L, in constant-rate feeding mode and single-dose feed-batch mode, respectively (Figure 5C). Additionally, productivity [1.06 g/(L/h)] was significantly enhanced when compared with results from a previous study (Table 3).

**TABLE 3:** Comparison of productivity of 2,5-DKG in different literatures.

Substrate	Fermentation time (h)	Productivity of 2,5-DKG	Fermentation mode	Source
100 g/L glucose	120	0.55 g/L/h	Batch fermentation, 26°C	Qazi et al. (1991)
50 g/L glucose	150	0.31 g/L/h	Mixed fermentation, 30°C	Sulo et al. (2001)
25 g/L glucose + 25 g/L GA	32	0.08 g/L/h	Resting-cell system, 28°C	Ji and Gao (2001)
100 g/L glucose	60	0.36 g/L/h	Batch fermentation, 30°C	This study
20 g/L glucose	16	0.77 g/L/h	Batch fermentation, 30°C	
100 g/L glucose (20 g/L glucose + glucose feeding)	48	1.06 g/L/h	Fed-batch fermentation, 30°C	

## Discussion

Fermentation broth containing 2,5-DKG is usually darker in color, and therefore, the detection method, which was based on NH_4_OH-HCl reactions (Sulo et al., 2001), caused a large deviation due to culture broth interference. This inevitably affected the absorbance and generated higher 2,5-DKG yields. HPLC approach was relatively accurate; however, a good standard was required to generate a standard curve as 2,5-DKG standards were commercially unavailable, and product purity from self-purification is low. So, there is still a large deviation in the HPLC detection method, resulting in that the detection value of 2,5-DKG in previous studies is much greater than its real value (Qazi et al., 1991). Based on this problem, the method of pure enzyme catalysis can effectively avoid the shortcomings of no standard or insufficient purity of standard, and more accurately quantify 2,5-DKG in the reaction system.

The chassis strain *G. oxydans* ATCC9937 screened by the new quantitative method has a strong membrane dehydrogenase system (Bringer and Bott, 2016; Zou et al., 2017; Jin et al., 2019; Zhou et al., 2019; Stefanie et al., 2021), only about 10% D-glucose is phosphorylated into the pentose phosphate pathway (PPP pathway) after entering the cell and is used for oxidative function and cell growth (Krajewski et al., 2010). Most D-glucose is oxidized in its periplasmic space (Dai et al., 2022) and the final product of oxidation is 2,5-DKG. Some strains cannot completely consume D-gluconic acid, resulting in the conversion of D-gluconic acid to 2-KG becoming the rate-limiting factor, but this situation did not exist in *G. oxydans* ATCC9937, which can completely consume the D-gluconic acid and 2-KG produced in the fermentation broth, but the final 2,5-DKG is less, and the conversion from 2-KG to 2,5-DKG is low.

The conventional idea of metabolic engineering is to directly overexpress the rate-limiting enzyme to improve the yield, but this operation is not conducive to the discovery of side effects. 2-KG can be completely consumed, but the yield of its oxidized product 2,5-DKG is very low. When the cause of this situation is unclear, it is very important to first verify whether there are side reactions and convert 2-KG into other substances. To find the speed-limiting factor more accurately, this study first knocked out the possible rate-limiting factor *kgdSLC*, and then verified its overexpression. However, the knockout and overexpression results of *kgdSLC* showed that the metabolic pathway of by-products of 2-KG did not seem to exist or was very few. 2-KG is a relatively stable compound, which can exist in the fermentation broth for a long time without other degradation reactions. The powerful periplasmic dehydrogenase system of *G. oxydans* ATCC9937 can ensure the continuous and efficient reaction of D-glucose to 2,5-DKG, overexpression of *kgdSLC* by genetic engineering could not further increase the yield of 2,5-DKG. 2-KDGH is not the main rate-limiting factor of the reaction, and the main speed-limiting factor should be the degradation of 2-KG. Many studies have mentioned that 2,5-DKG is easy to degrade, but no research has proved how it is degraded. The data provided in this study showed that the main reason for the low yield of 2,5-DKG is its continuous degradation in the fermentation broth, and the main mode of degradation is browning.

Many substances will be browning during storage (Tan et al., 2020; Fatouros et al., 2021). Browning mainly includes enzymatic and non-enzymatic two pathways (Paravisini and Peterson, 2019; Pham et al., 2020). The browning of 2,5-DKG is mainly through non-enzymatic reaction. This color change was similar to L-ascorbic acid browning, which is caused by a series of complex reactions during its degradation (Paravisini and Peterson, 2019), but is primarily caused by 2,3-DKG (2,3-diketo-L-gulonic acid) production (Forouhar et al., 2004). The hydration of 2,3-DKG occurs during degradation and generates colored substances (Penney and Zilva, 1945). As 2,5-DKG contained two carbonyls (Figure 4D), it may have had similar hydration reactions to 2,3-DKG, thereby producing colored substances. Previous studies suggested that 2,3-DKG may have similar antioxidant properties to L-ascorbic acid (Li et al., 2001). Whether 2,5-DKG is also prone to browning, due to its strong reductive capabilities, warrants further research. Therefore, using color deepening as a standard for 2,5-DKG generation is inaccurate. Thus, 2,5-DKG had no color, whereas a yellowish-brown color was caused by browning. Importantly, this browning required no specific temperature; even at 30°C, 2,5-DKG continued browning with increased content and fermentation time. Because the substances produced by browning are complex, we have not analyzed the detailed browning products for the time being, which may be targeted in subsequent studies. Moreover, the browning phenomenon was irreversible; the only way to slow down the process was to shorten fermentation times and accelerate substrate conversion to 2,5-DKG.

In previous studies, fermentation 2,5-DKG usually takes more than 120 h. However, continuous aeration and stirring during fermentation will further accelerate the browning of 2,5-DKG. Too long fermentation time leads to a large amount of browning of 2,5-DKG in the fermentation broth, resulting in very low actual purity of 2,5-DKG. Although the powerful dehydrogenase system enables to oxidize sugar alcohols in the reaction system without excessive biomass (Zhou et al., 2019; Han et al., 2021), too high initial sugar concentration will still inhibit its growth rate and corresponding enzyme activity, resulting in the increase in reaction time. By adjusting the initial D-glucose concentration in the broth, cell growth and substrate transformation efficiency were both accelerated. The time of bacterial transformation of substrate was also greatly shortened, the significant shortening of fermentation time reduced the browning rate of 2,5-DKG in the medium and greatly increased the final yield of 2,5-DKG. Browning will not only affect the accumulation of 2,5-DKG but also the dark substances produced by browning will stain the cell membrane, which will seriously affect the construction of the subsequent 2,5-DKG to 2-KLG pathway. Further research is required to analyze the main mechanisms underpinning 2,5-DKG browning, verify whether there are metabolites easy to combine with it to cause browning in the fermentation process, and further transform the metabolic pathway.

## Data Availability

The original contributions presented in the study are included in the article/Supplementary Material; further inquiries can be directed to the corresponding author.
