# Supplementary material for: Efficient Production of 2,5-Diketo-D-gluconic Acid by Reducing Browning Levels During Gluconobacter oxydans ATCC 9937 Fermentation
Source: Front Bioeng Biotechnol. 2022 Jul 8;10:918277. doi: 10.3389/fbioe.2022.918277 (PMC9304662; doi:10.3389/fbioe.2022.918277)
Supplement: Supplementary file 2 [file Table1.DOCX]

kgdSLC:

GAAATGACAATGATCTCTGTTGCGGAACATCGTGGAAAAGGAGCCAAGGCATGACCAAAGGCCGCCATCCTTCCGCTCCTGTTCAGCAGTCTGTCCTGGGACGCCGCTCCCTGCTGCTCGGCACCATCATGGGCAGTATTTCTGTTGCTGTCGGCAATTCAGTCCTTGGAAAGGCAAGCGCTCTTGCCGCCACTCCACTCGATGAAACATTCCTGGCCATCTCACGGGCGATCACCGGGCGTCAGGATCTTGATCCAATCCTCAGCAGCCGCCTCTGCGCGTCCATGCAGGCCACTTTTCCTGGCTATGCAAGCACAATCCAGGCCCTGGCCTCTGTCACGGCGCAAGGGGGGCAGCCCCGCGAAATTCTCGAGCGCTCCGGCGATCTGAAAAAGGCTTTTCTGGCCCTCAATGCCGCCTGGTACACCGGCTCAGTCGAGGACAGAACGGGCGCCCCCATGGTCGCCTATTACAATGCGCTCATGTACCAGCCCACGAAAGATGGCCTGCCGGTACCAACCTACTGCTTTGCACGTCCAGGATGGTGGACCGAGACACCGCCGGCCCTCGGCATTCCGATCCATGCTCCGCTTCCGGCGACGCCACCGCCCCCGGCTCCTCTCGGCGTGGAAAGCAAAACTCCGCCGCAGTCGCCGCCCCTGAAACCCATCACGCCCTCTTCTCCCCCCTACGTTCCCAAACCCCGTCAGGAGCACTAACCCATGGCCAAACCGGACTTTTCATCTTCTGGAGATGTATCGGCGGATGTTGTTGTCGTCGGCTCCGGAGTGGTGGGCGGCATGGTGGCAAACGAACTTGCTGCTGCTGGCCATTCCGTACTGATTCTGGAGGCAGGACTGCGCCTCAAACGCGCAGAATATGTGGAAAACTGGCGCAATATGTCGTTCCACAACCGGCTTCATTCTGATTTTCAGGGGCTTTATCCCCAGTCCCCGCTGGCTCAGGCCCCCTTATATTTTCCGCCCAATGACTATGTAGGCCTGTCGGGCCCCAATGGCTCGGCGTTCAAGCAGGGCTATCTGCGCACCGTCGGCGGAACGACATGGCACTGGGCCGCTTCCTGCTGGCGTCATCTTCCTTCCGATTTCGAGATGAAAACGCGTTATGGCGTCGGCCGCGACTGGGCCATCTCCTACAATGAGCTGGAACCGTTTTATTGCCGCGCTGAAGAGGAAATGGGCGTTTCAGGACCGCACGACAAATCTCTCCAGTCCCCTCCGGAACGGAGCCGCCCCTACCCCCGGGACATGATCCCGTTCGGGTATGGAGACCGTCGGGTCGCTGAAATCGTCAATCCCCACGGTTTTCATCTCGTTCCCATTCCACAGGGACGGAGTATCGAGCCTTACGGTGAGCGCCCCGCCTGCTGTGGCAATAACAACTGCCAGCCCATCTGCCCCATCGGGGCCATGTACAATGGCATTCAGCACATCGAAAAAGCTGAGTCCAAAGGCGCTGTCACCATCGCCGAAGCGGTCGTCTATCGTATTGATACCGATGAAAACAACCGCGTGACGGCCGTGCACTGGTATGACGCGCACCGGCAGTCCCACAAGGCGACCGGCAAGACATTCGTGCTCGCCTGTAATGGTATCGAAACCCCGCGTCTGCTGCTGCTGGCGGCGAATGAACGCAACCCGAATGGCATCGCCAACTCCTCGGATCAGGTGGGCCGCAACATGATGGACCACTCGGGCGTCCATGCATCCTTTCTGGCCAACGAGCCTCTCTGGTTCGGACGCGGACCAGCGCAGAGCAGCTGCATCGTCGGCCCCCGGGACGGAGATTTCCGGCGGGAATACTCGGCCAACAAGATGATCCTCAACAATATCAGCCGCGTCGCTCCTGCGACGGAACAGGCCCTCAAACTGGGTCTGGTCGGCAAAGAGCTTGATGACGAAATCCGCCGCCGTGCAGCATGCGGTGTTGACCTCTCCATCAGCCTTGAACCGCTTCCTGATCCGGAAAATCGCCTGACGCTGAGCAAAACCCGCAAGGATCCGCTGGGGCTGGCCTGCCCCGACATCCATTACGATGTCGGCGACTATGTCCGCAAAGGACTGGATGCGGTCAACACGCAGGTCAAGCAGATTGCCGGTCTGCTGGGTGGCACCGAACTCCGTATCACGACGGACTATAACGCCAATAATCATATCATGGGCGGTACGATCATGGGGCGGGACCCCAAGGACTCGGTCGTAGACGGAGACTGCCGGGCCCATGATCACCCCAATCTGTGGATTCCCGGCGGCGGCGCGATGCCGTCTGCCAGCGTCGTCAACACCACGCTCAGCATGGCAGCGCTCGGTCTGAAAGCTGCCGATTCCATCAAGGCCGCTTTCGCACGGGGGCAGGCATGAAACAGAAACGACAGAAAAGCCTGGGTCTGCCCGGCTATTTCGCCCTTTCCGCTGCAATCGGACTTGGAACCGGCACATTTGCGCATGCGCAGGACACTGATCCCAACACGGCTGTCGCCGAACGCGGACATTATCTTGCCATCGCCGCTGACTGCGCGGCCTGCCACACGGCGCCCGGCTCCAGCAAAGCGTTTGCCGGCGGGTATGGCATCGCGTCTCCACTTGGATCGATCTATTCGACCAACATCACGCCTTCAAAAGAATACGGCATCGGGAACTACACCGAGAAAGAGTTCGCCCATGCGTTACGGGAAGGCATCCGACGCGACGGGGCTCATCTTTACCCCGCGATGCCTTACACCTCGTACACCAAGCTGACAGACGACGACGTCCACGCCCTGTACGTCTATTTCATGACATCGGTGAAACCGGTCGAAAGCAGCCCGCAGAAAACCGAGCTACCTTTCCCCTATAATATCCGGGCTTCCATGGCGATCTGGAATGCCCTTTTCCTGGATGATACCCGCTTCAAGCCCGATCCTTCCAAATCCGTGGAAGTCAATCGCGGGCATTACCTGTCCTACGCCCTGGCCCATTGTGACACCTGCCACACGCCCCGCAACGCCATGATGGCGGAGAAGGGAAGCAGCCCCCTGGCCGGTGCGTCGCTGTCCTCATGGTATGCGCCGAATGTGACTTCGGACCCTGTCAGCGGTATTGGCGGCTGGAGCAACGACGATCTTTTCCGCTACCTCAAAACCGGCGATGTTCCCGGAAAAGCTCAGGCAGGCGGCCCCATGGCAGAAGCCATCGAGCACAGCTTCCAGTATCTGAGCGATGCCGACATCCGTGCCATGGTTGCTTACATCAAGCAGGTTCCGGCCATTTCGGACAGCAAGGACAAAGCCCCCCGCGAAAGCTATGGGAAAATTTCCGATCGTGAATCCGTCTACCGCGCTCTGCCCGCCAGCCGGATCAGTCGCGGCGAATATCTCTTCTCGGGAGAGTGTGCGGCCTGCCACCGCCCAACCGGCCAGGGCAGCGCGGACGGGTACTACCCACAGCTTTTCCATAATACGGCCCTGGGCGCCCCGATCGCCGATAACCTGATCACTACGATCCTCATGGGGGTTCGGCGGGAGGTCAACGGTCATACGACCTATATGCCTGGATTTGGGCCAGGTTCGTATGTGGACTCCCTGTCCGATCAGGACATTGCCGATATCAGCAATTACGTTGAGCAGCGCTTCGGCAATCCGAATGTCAAAGTCACACCTGACGATGTGAAGCTCATCCGTTCCGGTGGTCCCAAGCCGCTTATTGCACAGCTGGGCGCTTTCACAGTCCCCGCGATGATCGGAGCAGCCCTGATCTTCGCCGTCGCCATTCTTTTCTTCATCCGACTTTCCCGAAAGGCGAAGGCCTGA

2,5-DKGR:

Atggatcagaagaataagctttcgaagtctgaaggtatcccattggtcaccttgaatgatggaaaaaccattcctcagcttggttttggtgtgttcaaggtagatcccgatgaagcagagcgcgtagttaccgaagcacttgaggtaggttaccgccacatcgatactgctgcgatttacggcaatgaggaaggtgtcggccgagctattgctaagtccggcattcctcgtgaagagctgtttattactaccaagttgtggaacgatcgccacctggatgtagaagctgcttttgaggagtctctgcagaagctgggcttggattatgtagatctgtacttggtgcactggccggcaccgaagaacgataattatgttgctgcatggaagggcttggaaaagctcggtgaccgtgctcgttccatcggtgtgtgcaacttcctgccagagcacctagaaaagctgctggcagaggcaaccactgtgcctgccattaaccagattgagctgcacccagctttgcagcagcgcgatgctgttgaggcatctcttgcagcaggcatcactgtggagtcgtggggtcctctgggacaggggcgttttgatcttggcgctgaggaaccaatcgcagctgcagcgaagaaccatggaaagaccccagctcaggttgttatccgttggcacctgcagaacggtttcgttgtgttccccaagactgtgactaagagccgcatggtggaaaacatcgacgtgtttgatttcgaactcagtgatgaggagatggctgcgatcactgctcttgagcgcaatgatcgtggtggttcacacccgaatgatctgaactag
