# Supplementary material for: Efficient Production of 2,5-Diketo-D-gluconic Acid by Reducing Browning Levels During Gluconobacter oxydans ATCC 9937 Fermentation
Source: Front Bioeng Biotechnol. 2022 Jul 8;10:918277. doi: 10.3389/fbioe.2022.918277 (PMC9304662; doi:10.3389/fbioe.2022.918277)
Supplement: Supplementary file 3 [file Table3.DOCX]

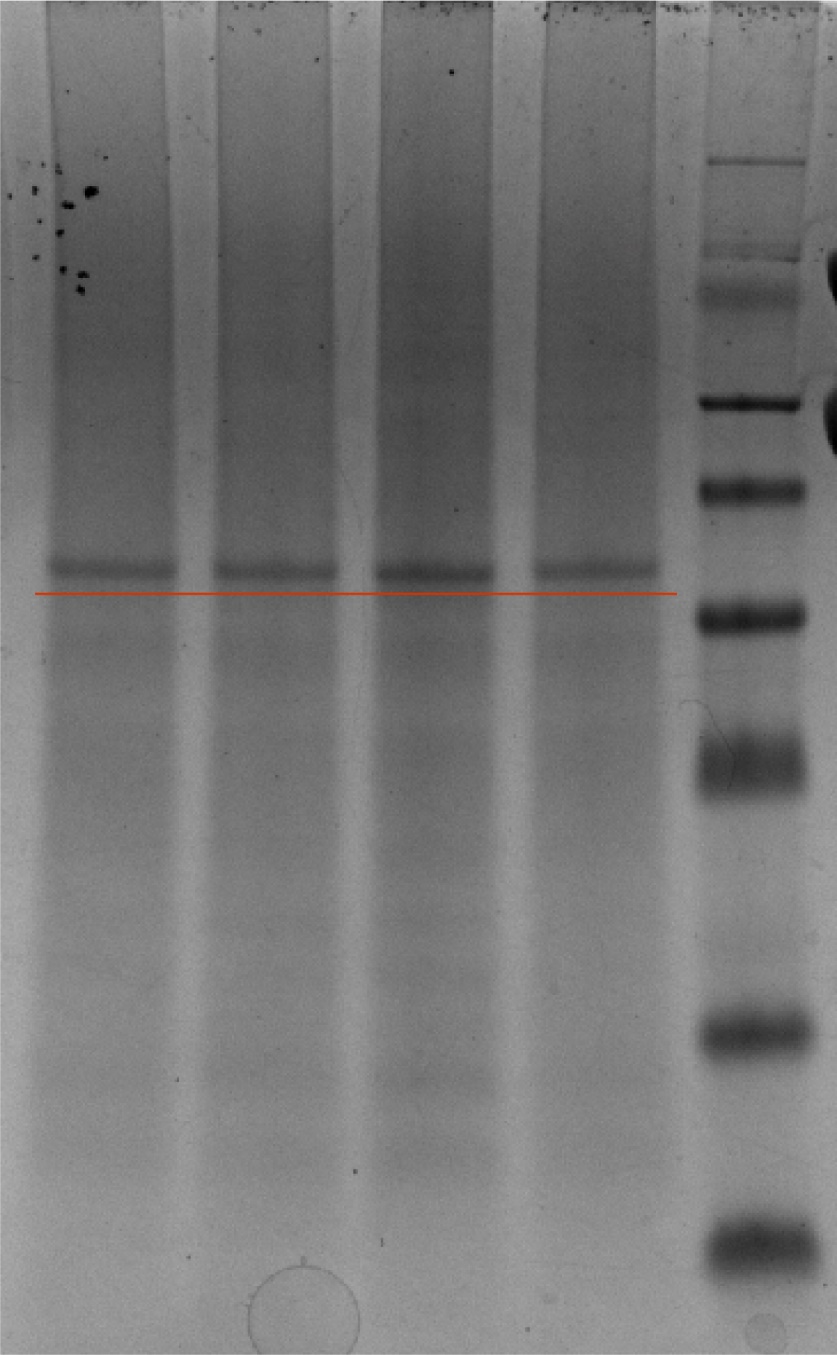


**Figure 1 SDS-Page of 2,5-DKG reductase**


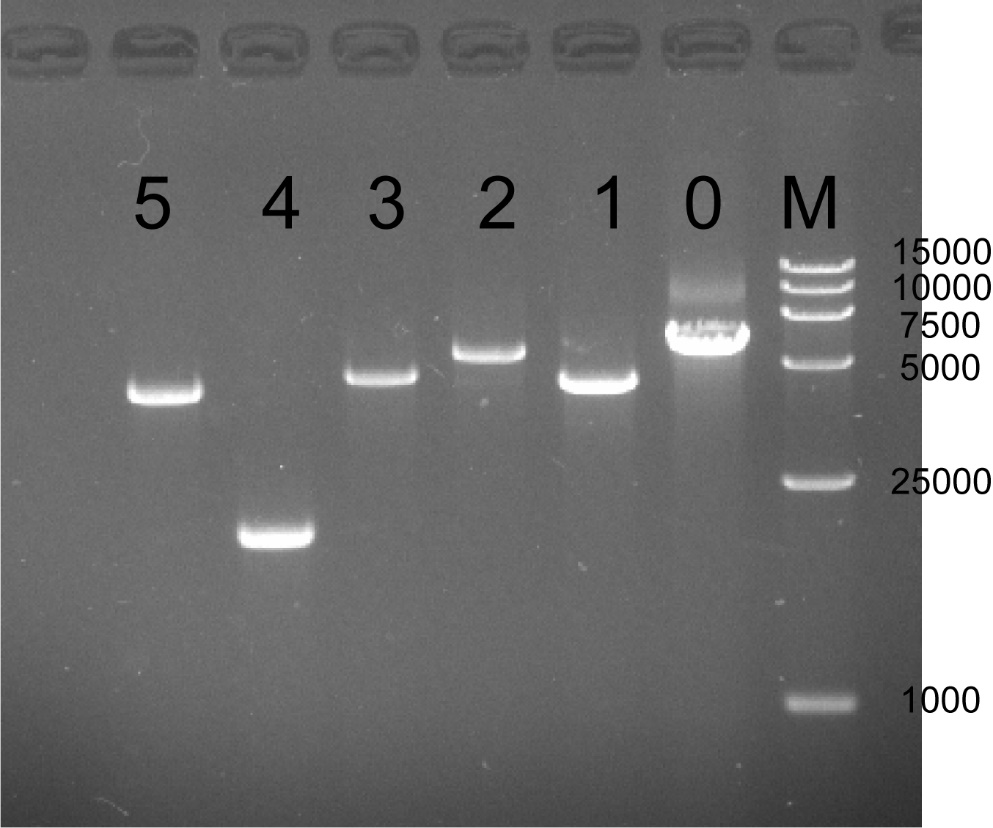


**Figure 2** ***kgdSLC* knockout strain:**

**M: Maker (15000bp); 0: control :1: knockout *kgdL*; 2: knockout *kgdS*; 3: knockout *kgdC*; 4: knockout *kgdSLC*; 5: Overexpression *kgdSLC***
