# Supplementary material for: Efficient Production of 2,5-Diketo-D-gluconic Acid by Reducing Browning Levels During Gluconobacter oxydans ATCC 9937 Fermentation
Source: Front Bioeng Biotechnol. 2022 Jul 8;10:918277. doi: 10.3389/fbioe.2022.918277 (PMC9304662; doi:10.3389/fbioe.2022.918277)
Supplement: Supplementary file 4 [file Table4.DOCX]

Figure 1. LC-MS results after converting 2,5-DKG solution to 2-KLG using 2,5-DKG reductase

Figure 2. 2,5-DKG was detected by LC-MS
